# Visceral Obesity as a Predictor of Postoperative Complications After Pancreaticoduodenectomy

**DOI:** 10.7759/cureus.35815

**Published:** 2023-03-06

**Authors:** Krishna Ramavath, Satish Subbiah Nagaraj, Manish Kumar, Niladri Mohan Raypattanaik, Divya Dahiya, Ajay Savlania, Cherring Tandup, Naveen Kalra, Arunanshu Behera, Lileswar Kaman

**Affiliations:** 1 General Surgery, Postgraduate Institute of Medical Education and Research, Chandigarh, IND; 2 Radiodiagnosis and Imaging, Postgraduate Institute of Medical Education and Research, Chandigarh, IND

**Keywords:** cross-sectional imaging, pancreaticoduodenectomy, postoperative complications, surgery, visceral obesity

## Abstract

Introduction: Obesity is associated with increased morbidity and mortality post surgery. The measurement of visceral obesity can predict postoperative outcomes after pancreaticoduodenectomy.

Methods: This is a prospective observational study. Visceral obesity was calculated by measuring the fat thickness in the retro-renal area by using a computed tomography scan. Visceral obesity was defined as retro-renal fat thickness (RRFT) of ≥ 2 cm. Patients were divided into two groups: Group-A (RRFT < 2 cm, non-obese) and Group-B (RRFT > 2 cm, obese). Demographic, clinical, and intraoperative variables were correlated with postoperative outcomes.

Results: Fifty-six patients were included in the study. Thirty-two patients were included in Group-A, and 24 patients were included in Group-B. The two groups had comparable outcomes. A total of 21 patients in Group-A (65.62%) and 17 patients in Group-B (70.83%) had comorbidities, including diabetes mellitus, hypertension, and coronary disease (p=0.680). American Society of Anesthesiologists (ASA) grading was comparable (p=0.927). BMI was also comparable (p=0.354). Type of pancreaticoduodenectomy, pancreatic texture, pancreatic duct diameter, and technique of pancreaticojejunostomy anastomosis were comparable. The mean operative time was longer in Group-B (362 ± 36.2 min vs. 298 ± 45.2 min) (p=0.001). Intraoperative blood loss was more in Group-B (312 ± 36.8 ml vs. 267 ± 23.7 ml) (p=0.001). The rates of postoperative pancreatic fistula and delayed gastric emptying were comparable (p=0.402 and p=0.134, respectively). The length of hospital stay was longer in patients in Group-B (p=0.004). There was one death in Group-B (obese group).

Conclusion: Visceral obesity is a risk factor for postoperative complications after a pancreaticoduodenectomy.

## Introduction

Obesity is an emerging lifestyle disorder affecting the population globally [1]. It is associated with cardiovascular, neurological, and endocrine derangements and the development of metabolic syndrome [2-4]. Obese patients, when subjected to surgery, suffer an increased rate of surgical complications [4, 5]. Complications may vary from surgical site infection (SSI) to mortality as a consequence of the associated systemic compromise [5].

Pancreaticoduodenectomy (PD) is one of the most complex intraabdominal surgical procedures. With the recent advances in perioperative care, the mortality after PD has declined significantly, with a rate of less than 3% in high-volume centers [6]. However, the morbidity rate is still very high, ranging from 27%% to 43% [6-8]. Postoperative pancreatic fistula (POPF) is one of the most common and dreaded complications after PD. The outcome of PD directly corresponds to the integrity of the pancreatico-enteric anastomosis. Various risk factors have been identified for the development of POPF after PD. Pancreatic texture, main pancreatic duct diameter, and associated comorbidities have been identified as risk factors for developing POPF [6-8]. Soft pancreas has been consistently associated with POPF after PD in many large series from high-volume centers. Soft pancreas is, in turn, associated with fatty pancreas and obese patients, especially with visceral obesity [9-13]. The high deposition of adipose tissue in the abdominal cavity around the viscera, mainly the omentum, mesentery, and retroperitoneum, is defined as visceral obesity. Visceral obesity is directly associated with the development of metabolic syndrome and its ill effects [9].

Visceral obesity can be measured preoperatively by using various dynamic imaging modalities, including computed tomography (CT) scans [14-17]. The retro-renal fat thickness (RRFT) measurement in CT scans is a good marker of visceral obesity [16, 17]. The estimation of visceral obesity by measuring the thickness of RRFT before PD may help in predicting postoperative complications.

Here, we are reporting our experience of PD in patients with visceral obesity in terms of immediate outcomes, including POPF, delayed gastric emptying (DGE), postpancreatectomy hemorrhage (PPH), and SSI. An RRFT of more than 2 cm on the CT scan was taken as a surrogate marker for visceral obesity.

## Materials and methods

This is a prospective observational study conducted in Postgraduate Institute of Medical Education and Research, Chandigarh, India. The institutional ethics committee's approval was obtained for the study (Reference NK/2419/MS/11052-53). All patients between 18 years and 80 years of age with malignant pancreatic and periampullary tumors were included. A total of 56 patients who met the inclusion criteria were enrolled in the study. After obtaining informed written consent, demographic data, a detailed clinical history, and physical findings, including BMI, were recorded. All the patients underwent biochemical, hematological, and coagulation workups. Relevant tumor markers were analyzed and recorded. All patients underwent ultrasonography (USG) and contrast-enhanced computerized tomography (CECT) for staging workup. Visceral obesity was calculated by measuring the visceral fat in the retro-renal area by using a cross-sectional CT scan of the abdomen after overnight fasting. It was performed in the supine position at the end of the normal exhalation.

RRFT was determined by measuring the distance between the left posterior renal capsule and the junction of the abdominal wall and paraspinal musculature at the level of the left renal vein. Areas falling inside the range of −190 Hounsfield units to −30 Hounsfield units were selected as fat tissue. Visceral obesity was defined as an RRFT of ≥ 2 cm on CT scans. Patients were divided into two groups: Group-A (RRFT < 2 cm, non-obese) and Group-B (RRFT > 2 cm, obese). These patients were staged, and the surgical procedure (PD) followed the standard oncological principles. The intraoperative variables and the postoperative outcomes were recorded, analyzed, and correlated with the visceral obesity level. POPF, DGE, and PPH were defined according to the International Study Group of Pancreatic Surgery (ISGPS) guidelines [18].

Statistical analysis

Statistical analysis was done by using IBM SPSS Statistics for Windows, Version 20.0 (Released 2011; IBM Corp., Armonk, New York, United States). Quantitative data were presented as mean ± SD, median, and interquartile range. Categorical variables were represented as percentages. Measures of the Kolmgorov-Smirnov test checked the normality of the quantitative data. A t-test was used for normally distributed data, whereas the Mann-Whitney test was applied for skewed variables. The chi-square test and Fisher’s exact test were used to compare categorical variables. A p-value of < 0.05 was considered significant.

## Results

A total of 56 patients were enrolled in the study, among whom 32 patients were included in Group-A (non-obese) and 24 patients were included in Group-B (obese). The mean ages of the patients were 57.9 years and 55.9 years in the respective groups. Twenty-one patients in Group-A had co-morbidities such as hypertension, diabetes mellitus, and coronary artery disease compared to 17 patients in Group-B (p=0.680). The American Society of Anesthesiologists (ASA) grading and the BMI distribution of the two groups were comparable (p=0.927 and p=0.354, respectively) (Table 1).

**Table 1:** Demographic and clinical data. ASA: American Society of Anesthesiologists; BMI: body mass index

Variables	Group-A (n=32)	Group-B (n=24)	p-value
Age (years), mean±SD	57.92*±*2.1	55.94*±*3.6	0.012
Males/Females	21/11	14/10	0.777
Comorbidities			
Hypertension	6	4	0.887
Diabetes	12	9	0.773
Coronary artery disease	3	4	0.680
ASA grading			
I	11	9	0.927
II	19	14	
III	2	1	
BMI			
18.5 – 24.9	14	2	0.354
25.0 – 29.9	16	14	
30.0 – 34.9	2	8	

The type of PD in terms of classical Whipple's PD (CWPD) and pylorus-preserving PD (PPPD) was similar (p=0.421). Intraoperative blood loss and operative time were significantly more in Group-B (p=0.001 and p=0.001, respectively). Pancreatic texture, main pancreatic duct diameter, and pancreaticojejunostomy (PJ) techniques were comparable between the two groups (Table 2).

**Table 2:** Intra-operative data. PD: pancreaticodudenectomy; PPPD: pylorus preserving pancreaticodudenectomy; MPD: main pancreatic duct; PJ: pancreaticojejunostomy

Variables	Group-A (n=32)	Group-B (n= 24)	p-value
Type of PD			
Classical PD	21	14	0.421
PPPD	11	10	
Operative time, mean±SD	298±45.2 min	362±36.2 min	0.001
Blood loss, mean±SD	267±23.7 ml	312±36.8 ml	0.001
Pancreatic texture			
Soft	14	13	0.103
Hard	18	11	
MPD diameter			
< 3mm	4	3	0.158
3-5 mm	20	18	
> 5 mm	8	3	
PJ anastomosis			
Duct to mucosa	28	21	0.680
Dunking invagination	4	3	

The postoperative outcomes were comparable in the two groups (Table 3). Overall, 21 patients (37.50%) developed POPF: 10 in Group-A (31.25%) and 11 in Group-B (45.83%) (p=0.402). Thirteen patients had grade A fistula, and seven patients had grade B fistula. One patient in the obese group developed a grade C fistula. Seventeen patients (30.35%) developed DGE: eight in Group-A and nine in Group-B (p=0.134). A total of 17 (30.35%) patients developed SSIs: seven in Group-A and 10 in Group-B (p=0.213). The length of hospital stay was 12 days in Group-A and 16 days in Group-B (p=0.004), which was statistically significant. There was one mortality in Group-B due to POPF. On the minimum 90 days follow-up, no other surgery-related morbidity or mortality was observed. 

**Table 3:** Postoperative outcome data. POPF: postoperative pancreatic fistula; DGE: delayed gastric emptying; PPH: postpancreatectomy hemorrhage; SSI: surgical site infection

Parameter	Group-A (n=32)	Group-B (n=24)	p-value
POPF	10 (31.25%)	11 (45.83%)	0.402
Grade A	7	6	
Grade B	3	4	
Grade C		1	
DGE	8 (25%)	9 (37.5%)	0.134
Grade A	5	4	
Grade B	3	5	
Grade C			
PPH	1	1	0.603
SSI	7 (21.87%)	10 (41.66%)	0.213
Length of hospital stay (days)	12	16	0.004
Mortality	0	1	0.155

## Discussion

Obesity has become a real health concern and is becoming a global epidemic [1, 2]. Among adults, obesity is defined as a BMI of 30 or above. Obesity is associated with metabolic syndrome. The diagnostic criteria for metabolic syndrome are reduced high-density lipoprotein cholesterol, high triglycerides, hypertension, diabetes, and a large waist circumference [1-3]. Patients with metabolic syndrome are more prone to develop cardiovascular, neurological, and diabetes-related complications [3].

Obese patients, when subjected to surgery, suffer an increased rate of surgical complications [4, 5]. Short-term postoperative complications ranging from SSIs to mortality have been reported [4, 5]. Increased fat and softness of the tissues cause excessive trauma, increased blood loss, and prolonged operative time, leading to an increased rate of complications [5]. The incidence of gastrointestinal cancers such as esophageal cancer, colorectal cancer, and pancreatic cancer, have been reported to be high in obese patients [4].

PD is one of the most complex intra-abdominal surgical procedures. With the recent advances in perioperative care, the mortality rate after PD has declined significantly, with a rate of less than 3% in high-volume centers [6]. However, the morbidity rate is still very high, ranging from 27% to 43% [6-8]. POPF is the most common and dreaded complication after PD. The outcome of PD directly corresponds to the integrity of the pancreatico-enteric anastomosis. Various risk factors have been identified for the development of POPF after PD. Pancreatic textures, main pancreatic duct diameter, and associated co-morbidities have been identified as risk factors for developing POPF [6-8]. Soft pancreas has been consistently associated with POPF after PD in many large series from high-volume centers [8-13].

A soft, fatty pancreas is especially seen in patients with high visceral obesity. A high deposition of adipose tissue in the abdominal cavity around the viscera, mainly the omentum, mesentery, and retroperitoneum, is defined as visceral obesity [9, 10]. Visceral obesity is directly associated with the development of metabolic syndrome and its ill effects. Increased morbidity after pancreatic surgeries has been reported in cases with high visceral fat and increased BMI [8-13]. Rather than BMI, visceral fat may be an independent risk factor for POPF after PD. Increased visceral fat may directly affect the surgical procedure and complicate it by lengthening operating time, increasing blood loss, and more tissue trauma [10-13]. Increased soft tissue and vascularity to the visceral fat leads to increased operative blood loss, longer operating time, raised postoperative inflammation, and a higher risk of anastomotic disruption [8-13]. In this study, there was significant blood loss and operative duration in the obese group. The incidence of POPF was not significant in the obese group, however, with one grade C fistula. 

The presence of increased visceral fat leads to local complications associated with the surgical procedure. The incidence of SSI was high in the obese group (41.66%) but was not statistically significant. The presence of thick abdominal fat causes seroma formation and secondary infection primarily due to vascular compromise of the perforating vessels supplying the abdominal wall. The length of hospital stay is prolonged in obese patients undergoing PD due to its increased complications [11-13]. In this study, the length of hospital stay was significantly longer in the obese group.

Moreover, obesity can lead to increased systemic complications, including cardiac, pulmonary, and metabolic complications, which can worsen the postoperative outcomes [11-13]. The occurrence of hypertension, diabetes, and coronary artery disease in the present study was higher in the obese group but was statistically insignificant.

Visceral obesity can be measured preoperatively by various dynamic imaging modalities, including CT scans [14-17]. RRFT measurement in CT scans is a good marker of visceral obesity [16,17]. Various preoperative conditions such as cholangitis, preoperative stent, pancreatitis, previous abdominal surgeries, and malnutrition may interfere with abdominal fat, but the RRFT may be consistent and easier to calculate. 

Routine estimation of visceral obesity by using RRFT before PD may help predict postoperative complications such as POPF. Hence mandating proper counselling and intraoperative measures such as stenting across pancreatico-enteric anastomosis, omental wrapping, glue reinforcements, and use of somatostatin analogue may be considered.

This study has several limitations. First, the number of patients included was few during the study period. Second, two types of surgical procedures, namely classical Whipple's PD and pylorus-preserving PD, were performed. The type of PD may have influenced the operative variables such as operating time and blood loss. Third, two different types of PJ techniques were performed, in the form of the duct-to-mucosa and dunking invaginations techniques, which may have influenced the postoperative fistula rate and outcome. 

## Conclusions

We conclude that postoperative complications after PD were more in patients with visceral obesity in terms of operative blood loss, operating time, and hospital stay. Preoperative assessment of visceral obesity by measuring the retro-renal fat by using a CT scan may predict the postoperative outcomes after PD.
